# Attachment anxiety and depressive symptoms in undergraduate medical students: The mediating role of emotion regulation strategies

**DOI:** 10.1007/s40037-022-00713-z

**Published:** 2022-05-19

**Authors:** Valentina Colonnello, Edita Fino, Paolo Maria Russo

**Affiliations:** Department of Experimental, Diagnostic and Specialty Medicine, University of Bologna, Bologna, Italy

**Keywords:** Medical students, Anxious attachment, Emotion regulation strategies

## Abstract

**Introduction:**

Several studies report that medical students are at high risk of depression. Despite the variability in students’ vulnerability to depression, the role of individual differences in depression risk among medical students has hardly been investigated. Studies outside of medical student populations have shown that individual differences in attachment style and emotion regulation participate in vulnerability to depression.

**Objectives:**

This study investigates to what extent medical students’ depressive symptoms are related to differences in students’ insecure attachment styles and their perception of reduced access to emotion regulation strategies.

**Methods:**

In a cross-sectional quantitative study, undergraduate medical students at the beginning of their second academic year completed online questionnaires measuring their attachment style, difficulties in emotion regulation, and depressive symptoms.

**Results:**

Out of the 390 medical students invited, 267 participated in the survey. Higher secure attachment was associated with fewer depressive symptoms. Medical students’ insecure attachment style and emotion dysregulation were significantly related to depressive symptoms. Difficulties in employing strategies to disengage from one’s own negative affect partly mediated the effects of two dimensions of insecure anxious attachment—need for approval and preoccupation with relationships—on depressive symptoms.

**Discussion:**

Anxious attachment style and limited access to emotion regulation strategies participate in medical students’ depressive symptoms. These findings highlight the need for acknowledging medical students’ attachment style and students’ perceived access to emotion regulation strategies for the early identification of and intervention programs for the risk of depression.

**Supplementary Information:**

The online version of this article (10.1007/s40037-022-00713-z) contains supplementary material, which is available to authorized users.

## Introduction

Medical education is seen as psychologically challenging, so it is not surprising that medical students report more psychological distress than do non-medical students and the general population [1–3]. Concerns about medical students’ mental health have thus informed policy-makers and education institutions, leading to programs aimed at meeting their psychological needs [4, 5]. Though individual differences in depression risk among medical students is an important factor in these programs, the early identification of those differences and the role they play in depression have received little attention.

Attachment theory offers a promising framework for the study of those individual differences. According to attachment theory, individuals who experience parenting as accepting and responsive are more likely to show a secure attachment style characterized by a sense of self as worthy and by the perception of others as reliable [6, 7]. This secure attachment style is associated with positive health conditions and an ability to adapt to stressful circumstances. In contrast, people who experience inconsistent, rejecting, or undependable parenting tend to develop insecure attachments, which are viewed along the two dimensions of avoidant and anxious attachments [6, 8]. Avoidant attachment style may be expressed through discomfort with closeness and a tendency to evaluate relationships as secondary to achievement; anxious attachment style may instead emerge as a need for others’ approval and a preoccupation with rejection [9–11]. Insecure attachment, in either of its dimensions, may open a gateway for depression [12].

The extent to which attachment style is related to depressive symptoms in medical students has been largely unexplored. However, such research could benefit programs aimed at promoting medical students’ mental health, especially given previous studies showing that attachment style is a key factor involved in medical students’ happiness [13] and their vulnerability to perceived stress [14].

Difficulties in employing emotion regulation abilities may be one of the mechanisms through which insecure attachment style increases the risk of depressive symptoms [15, 16]. There is substantial evidence linking secure attachment with effective self-regulation of one’s emotions and insecure attachment with emotion dysregulation. In individuals with anxious attachment, such dysregulation is characterized by hyperactivating strategies, such as a persistent seeking of social proximity and comfort, whereas in individuals with avoidant attachment, it is characterized by deactivating strategies that are used to repress one’s emotions and to withdraw from closeness [6].

Though relatively scarce, emerging evidence suggests that emotion regulation partly mediates the relationship between attachment and depressive symptoms in student samples. For example, Owens and colleagues [17] showed that both attachment anxiety and attachment avoidance are positively associated with difficulties in emotion regulation strategies in undergraduate psychology students who experienced traumatic life events. Interestingly, they also found that emotion regulation difficulties affect the association between insecure attachment style and depression.

In summary, though students’ mental health is a key concern in medical education, research investigating the relationship between attachment, emotion regulation, and depressive symptoms among medical students is lacking. Our study therefore aims to address this gap by investigating the relationship between individual differences in attachment style and depressive symptoms and the possible mediating role of difficulties with emotion regulation strategies, that is, with students’ perceived lack of access to emotional resources. The perception of having reduced access to emotion regulation strategies may be one path to the feeling of hopelessness, a common characteristic of depression.

## Methods

### Participants and procedure

Participants were medical students at the beginning of their second academic year of the Medicine and Surgery Degree Program at the University of Bologna’s School of Medicine, a top-ranked university for medicine in Italy. By the end of their second year, medical students report psychological distress with a prevalence of moderate to severe depression [18]. We therefore recruited students enrolled in their second academic year. At the time of the data collection, students had just completed the first-year exam period and were adjusting to the beginning of the new academic year.

In November 2019, all 390 medical students were invited to complete an online survey consisting of questions about sociodemographic characteristics (sex, age, ethnicity, and years of education) and measures of attachment style [11], emotion regulation difficulties [19], and depressive symptoms [20]. The questionnaires were validated for the Italian population. All participants gave informed consent and were fully debriefed at the study’s conclusion. The procedures were approved by the University of Bologna institutional review board (IRB no. 273088).

### Instruments

Attachment was measured using the Attachment Style Questionnaire (ASQ) [10, 11]. The ASQ is a 40-item questionnaire exploring five dimensions of attachment: (a) secure attachment/confidence (*“I feel confident about relating to others”*); (b) avoidant attachment in its two dimensions of discomfort with closeness (*“I worry about people getting too close”*) and relationships as secondary to achievement (*“Doing your best is more important than getting along with others”*); and (c) anxious attachment in its two dimensions of preoccupation with relationships (*“I wonder how I would cope without someone to love me”*) and need for approval (*“I worry that I won’t measure up to other people”*). Responses were recorded on a 5-point Likert scale (1 = strongly disagree to 5 = strongly agree). The Cronbach’s alpha coefficients ranged between 0.65 and 0.70.

Difficulties in emotion regulation were measured using the Difficulties in Emotion Regulation Scale (DERS), which is a 36-item questionnaire with responses recorded on a 5-point Likert scale (1 = almost never to 5 = almost always); higher scores reflect greater emotion regulation difficulties [19, 21]. The DERS yields a total score (ranging from 36 to 180) as well as six scores for the following subscales: difficulty engaging in goal-directed behavior (Goals, *“When I’m upset, I have difficulty focusing on other things”*), impulse control difficulties (Impulse, *“When I’m upset, I become out of control”*), nonacceptance of emotional responses (Nonacceptance, *“When I’m upset, I become embarrassed for feeling that way”*), lack of awareness of emotions (Awareness, *“I pay attention to how I feel”*, reverse item), lack of emotional clarity (Clarity, *“I have no idea how I am feeling”*), and limited access to strategies for regulation (Strategies, *“When I’m upset, I believe there is nothing I can do to make myself feel better”*). The Cronbach’s alpha coefficients ranged between 0.80 and 0.90.

The Zung Self-Report Depression Scale (ZSDS) [20] is a 20-item questionnaire that uses a 4-point Likert scale to explore the frequency of depressive symptoms (e.g., *“I find it easy to make decisions”*). The Cronbach’s alpha coefficient was 0.80.

### Statistical analysis

The association between the attachment dimensions and depression and between these variables and the mediator (difficulties in emotion regulation) were explored using Pearson’s correlations.

To test the role of emotion dysregulation in mediating the relationship between attachment styles and depression, separate multiple mediation model analyses were performed using PROCESS macro for SPSS (Model 4, 5000 bootstrap resampling) [22, 23]. Controlling for the factor gender, total, direct, and indirect path coefficients were computed using standardized values. To test the significance of the indirect effects we examined, 95% confidence intervals were computed for the upper and lower potential limits following guidelines proposed by Shrout and Bolger [24].

## Results

Of the 390 invited medical students, 267 completed the survey. The students (151 women, 116 men, age: *M* = 20.52, *SD* = 2.48, European Credit Transfer and Accumulation System (ECTS): *M* = 51.14, *SD* = 10.13) were all Caucasians.

As illustrated in Tab. 1, all attachment dimensions were associated with depressive symptoms and emotion regulation difficulties, and emotion regulation difficulties were associated with depressive symptoms.

**Table 1:** Mean (*SD*) and intercorrelations (*r*) between the Attachment Style Questionnaire (ASQ), the Difficulties in Emotion Regulation Scale (DERS), and the Zung Self-Report Depression Scale (ZSDS)

		M (SD)	1	2	3	4	5	6	7	8	9	10	11	12
	*ASQ*													
1	*Confidence*	29.76 (5.31)	–	−0.67	0.38*	−0.52*	−0.26*	0.22*	−0.16*	−0.18*	−0.39*	−0.18*	−0.37*	−0.48
2	*Discomfort with Closeness*	37.16 (7.64)	−0.67″	–	0.55*	0.38*	0.16	−0.11	0.16*	0.19*	0.36*	0.24*	0.33*	0.36*
3	*Relationships as Secondary*	16.60 (4.70)	−0.38*	0.55*	–	0.23*	0.02	−0.19	0.06	0.′13″	0.20″	0.21″	0.27″	0.50*
4	*Need for Approval*	21.17 (5.63)	−0.52*	0.38*	0.23*	–	0.51*	−0.08	0.39*	0.31*	0.50*	0.33*	0.54*	0.35*
5	*Preoccupation with Relationships*	30.13 (5.57)	−0.26*	0.16	0.02	0.51*	–	0.15	0.30*	0.28*	0.31*	0.21*	0.42*	0.26*
	*DERS*													
6	*Awareness*	21.95 (4.58)	0.22*	−0.11	−0.19	−0.08	0.15	–	0.14	0.14	−0.08	−0.22	0.06	−0.20*
7	*Goals*	15.51 (3.68)	−0.16*	0.16*	0.06	0.39*	0.30*	0.14	–	0.52*	0.36*	0.26*	0.58*	0.34*
8	*Impulse*	13.10 (3.85)	−0.18*	0.19*	0.13″	0.31*	0.28*	0.14	0.52*	–	0.39*	0.26*	0.63*	0.42*
9	* Nonacceptance*	14.12 (6.34)	−0.39*	0.36*	0.20″	0.50*	0.31*	−0.08	0.36*	0.39*	–	0.23*	0.63*	0.50*
10	*Clarity*	11.28 (2.65)	−0.18*	0.24*	0.21″	0.33*	0.21*	−0.22	0.26*	0.26*	0.23*	–	0.33*	0.44*
11	*Strategies*	19.82 (5.87)	−0.37*	0.33*	0.27″	0.54*	0.42*	0.06	0.58*	0.63*	0.63*	0.33*	–	0.57*
12	**ZSDS**	38.01 (7.99)	−0.48*	0.36*	0.50*	0.35*	0.26*	−0.20*	0.34*	0.42*	0.50*	0.44*	0.57*	–

*p < 0.05, **p < 0.01

The multiple mediation model analysis showed a significant effect of attachment style on depressive symptoms. Specifically, higher secure attachment (confidence) was associated with fewer depressive symptoms (β = −0.47, 95% CI [−0.86, −0.54], *p* < 0.001), whereas the dimensions of higher discomfort with closeness (β = 0.36, 95% CI [0.26, 0.49], *p* < 0.001), perception of relationships as secondary to achievement (β = 0.27, 95% CI [0.27, 0.66], *p* < 0.001), need for approval (β = 0.49, 95% CI [0.55, 0.84], *p* < 0.001), and preoccupation with relationships (β = 0.33, 95% CI [0.31, 0.64], *p* = 0.019) were associated with more frequent depressive symptoms.

**Fig. 1:**
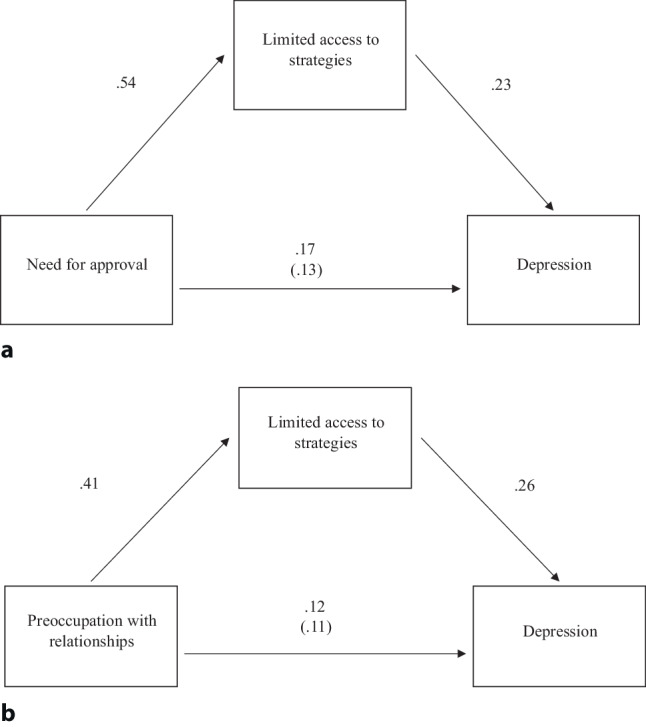
The mediating role of limited access to strategies in the relationship between depression and **a** need for approval and **b** preoccupation with relationships. Standardized coefficients of direct and indirect (*in brackets*) effects are presented

With respect to the mediating role of emotion dysregulation, indirect effects (coefficient ≥ 0.10) revealed that Strategies was the only category of emotion regulation difficulties that mediated the effect of attachment style on depressive symptoms. This partial mediation was specific to the two anxious attachment dimensions: need for approval (β = 0.13, 95% CI [0.05, 0.21]) and preoccupation with relationships (β = 0.11, 95% CI [0.05, 0.18]; Fig. 1). It was negligible (β < 0.10) for the other attachment dimensions. The mediating effect of other aspects of emotion regulation difficulties on the relationship between attachment style and depressive symptoms was also negligible (zero was included in the 95% CI). The total, direct, and indirect effects and the related CI intervals of the relationship between attachment dimensions, emotion dysregulation aspects, and depressive symptoms are presented in Table S1 in the Electronic Supplementary Material (ESM).

## Discussion

Medical education systems grapple with depression and psychological health issues in their students [1, 2, 14]. To better understand the factors involved in the risk of depression among medical students, investigations into the role of individual differences are crucial. In our study, individual differences in attachment style were related to students’ depressive symptoms: the higher the students scored on confidence (secure attachment), the less they reported experiencing depressive symptoms; however, the higher they scored on insecure attachment, the more they reported depressive symptoms. This result is consistent with previous findings indicating that secure attachment is associated with mental health and that insecure anxious attachment predicts poor health conditions [16, 17].

Our findings also highlight the key role of strategy difficulties (i.e., the feeling of not being able to successfully regulate one’s own negative affects). Though the other aspects of emotion dysregulation did not substantially mediate the relationship between attachment and depression, emotion regulation strategy partly mediated the association between depression and attachment anxiety in its two dimensions of need for approval and preoccupation with relationships. This finding agrees with previous studies indicating that depressive symptoms may emerge from a sustained hyperactivation of the emotional system involved in separation distress [25], and it resonates well with studies suggesting that anxious attachment is characterized by less perceived control of emotionally relevant stimuli, especially negative and social stimuli [26].

In addition, our findings corroborate previous studies showing that emotion dysregulation relates to depression [17, 27]. Presumably, through the perceived limited access to successful coping strategies, a student’s preexisting insecure attachment increased their perception of highly demanding medical training as a threat and exacerbated their self-reported depressive symptoms.

Our finding showing a relationship between attachment, depression, and emotion regulation in medical students agrees with Thomson and colleagues’ [14] findings showing that resilience, that is, the ability to thrive under stress, partly mediates the relationship between attachment and perceived stress in a medical student population. Our study is not exempt from limitations. Specifically, the study population was homogeneous in age, which limits the generalizability of our findings. Additional limitations are the sole use of self-reports not substantiated by actual clinical assessment and the auto-selection bias of respondents, with about 30% of the invited students declining to participate.

A further limitation is inherent in the study’s correlational nature, which does not lead to identifying a causal relationship between factors and thus leaves the findings open to alternative conclusions. For example, stressful situations such as highly demanding medical training may elicit insecure attachment patterns and increase the perception of not being able to access personal coping strategies.

Of note, though items in the DERS and ZSDS scales do not overlap, some facets of the DERS relate to depressive symptoms. For example, the facet of limited access to the strategies of emotion dysregulation, as conceptualized by Gratz and Romer [19], is evocative of depressive symptoms, such as hopelessness and rumination, as expressed, for example, by the DERS items “*When I*’*m upset, I believe that I will remain that way for a long time*” and “*When I*’*m upset, I believe that wallowing in it is all I can do.*”

Despite these limitations, to our knowledge, this is the first study indicating that individual differences in anxious attachment style and emotion regulation ability participate in medical students’ vulnerability to depression. Our findings on the importance of perceived difficulties in emotion regulation strategies in medical students offer a basis for further investigations into the specific emotion regulation strategies, such as reappraisal, suppression, and acceptance, that medical students commonly use to cope with stressful times in their medical training.

### Implications for medical education

Our findings also contribute to providing practical suggestions for curriculum development. For example, sustaining secure attachment, while weakening the role of anxious and avoidant attachment styles, and improving medical students’ emotion regulation abilities might be possible and desirable goals of education systems concerned with students’ well-being.

Both attachment style and emotion regulation ability are amenable to changes through life experiences and significant social relationships [28]. In adolescence and adulthood, for example, secure attachment may be boosted through relationships with peers and role models [28, 29]. Thus, enhancing medical students’ secure attachment, a protective factor against depressive symptoms, might be possible by providing social learning experiences that foster feelings of safety and worthiness, such as peer-learning programs and modeling-based training.

Additionally, given that medical training unavoidably encompasses experiences of stress, fears of failure, and hopes for remediation [30, 31] that require emotion regulation abilities, it may be useful/valuable to promote curricula that enhance students’ ability to focus and reflect on their own emotions and emotion regulation strategies. Some have even suggested that interventions aimed at improving emotion regulation ability may contribute to reducing the risk of depressive symptoms [2].

The professional medical curriculum might therefore be strengthened by including psychology courses designed to promote students’ awareness of their own individual characteristics and attachment-related affective resources. Future studies should specifically test the efficacy of proposed interventions in increasing secure attachment affective-cognitive resources and, in turn, in reducing depression risk among medical students. In addition, future studies should extend the scope of the present study by exploring the link between medical students’ attachment style, academic motivation and ability to empathize with patients, especially considering that the activation of a confident and secure caring mindset enhances medical students’ motivation toward learning material [32] and their ability to recognize others’ emotions [33].

Finally, our findings suggest the need for a concerted policy strategy to promote medical curricula that consider individual differences in the various facets of insecure attachment and vulnerability to depression. Such programs should also recognize the profound value of nurturing medical students’ affective resources and strengthening their ability to access effective emotion regulation strategies.

## Supplementary Information

**Table S1** Total, direct and indirect effects of the multiple mediation models examining the role of emotion dysregulation (DERS) on the relationship between the anxious attachment dimensions, Need for approval (a) and Preoccupation with relationships (b), with depression (ZSDS) *p < 0.05, **p < 0.01.
