# Supplementary material for: Attachment anxiety and depressive symptoms in undergraduate medical students: The mediating role of emotion regulation strategies
Source: Perspect Med Educ. 2022 May 19;11(4):207–12. doi: 10.1007/s40037-022-00713-z (PMC9391533; doi:10.1007/s40037-022-00713-z)
Supplement: Supplementary file 1 — Table S1 Total, direct and indirect effects of the multiple mediation models examining the role of emotion dysregulation (DERS) on the relationship between the anxious attachment dimensions, Need for approval (a) and Preoccupation with relationships (b), with depression (ZSDS) *p < 0.05, **p < 0.01. [file 40037_2022_713_MOESM1_ESM.docx]

**Electronic Supplementary Material**

**Table S1** Total, direct and indirect effects of the multiple mediation models examining the role of emotion dysregulation (DERS) on the relationship between the anxious attachment dimensions, Need for approval (a) and Preoccupation with relationships (b), with depression (ZSDS) **p* < .05; ** *p* < .01.

| **a)** | Model Pathway: Need for approval -> Emotion Dysregulation -> Depression | *β* | *Bias-corrected 95% CI* | | |  | *p* |  | R^2^ = 0.49 |
| --- | --- | --- | --- | --- | --- | --- | --- | --- | --- |
|  | Direct Effects |  |  |  |  |  |  |  |  |
|  | Need for approval -> Non acceptance | 0.49 | 0.44 | ─ | 0.67 |  | ** |  |  |
|  | Need for approval -> Goals | 0.40 | 0.19 | ─ | 0.34 |  | ** |  |  |
|  | Need for approval -> Impulse | 0.32 | 0.14 | ─ | 0.30 |  | ** |  |  |
|  | Need for approval -> Awareness | -0.08 | -0.17 | ─ | 0.03 |  | NS |  |  |
|  | Need for approval -> Strategies | 0.54 | 0.46 | ─ | 0.67 |  | ** |  |  |
|  | Need for approval -> Clarity | 0.34 | 0.10 | ─ | 0.21 |  | ** |  |  |
|  |  |  |  |  |  |  |  |  |  |
|  | Need for approval -> Depression | 0.17 | 0.09 | ─ | 0.40 |  | ** |  |  |
|  | Non acceptance -> Depression | 0.15 | 0.04 | ─ | 0.33 |  | * |  |  |
|  | Goals -> Depression | -0.01 | -0.26 | ─ | 0.23 |  | NS |  |  |
|  | Impulse -> Depression | 0.14 | 0.04 | ─ | 0.53 |  | * |  |  |
|  | Awareness -> Depression | -0.17 | -0.47 | ─ | -0.14 |  | ** |  |  |
|  | Strategies -> Depression | 0.23 | 0.12 | ─ | 0.52 |  | ** |  |  |
|  | Clarity -> Depression | 0.20 | 0.30 | ─ | 0.90 |  | ** |  |  |
|  | **Total Effect** | **0.49** | **0.55** | ─ | **0.85** |  | 0.001 |  |  |
|  | Indirect Effects |  |  |  |  |  |  |  |  |
|  | **Total Indirect Effect** | **0.32** | **0.24** | **─** | **0.41** |  |  |  |  |
|  | Need for approval -> Non acceptance -> Depression | 0.07 | 0.01 | ─ | 0.15 |  |  |  |  |
|  | Need for approval -> Goals -> Depression | 0.00 | -0.05 | ─ | 0.04 |  |  |  |  |
|  | Need for approval -> Impulse -> Depression | 0.04 | 0.00 | ─ | 0.09 |  |  |  |  |
|  | Need for approval -> Awareness -> Depression | 0.01 | -0.01 | ─ | 0.04 |  |  |  |  |
|  | **Need for approval -> Strategies -> Depression** | **0.13** | **0.05** | **─** | **0.21** |  |  |  |  |
|  | Need for approval -> Clarity -> Depression | 0.07 | 0.02 | ─ | 0.12 |  |  |  |  |

| **b)** | Model Pathway: Preoccupation with Relationships -> Emotion Dysregulation -> Depression | *β* | *Bias-corrected 95% CI* | | |  | *p* |  | R^2^ = 0.48 |
| --- | --- | --- | --- | --- | --- | --- | --- | --- | --- |
|  | Direct Effects |  |  |  |  |  |  |  |  |
|  | Preoccupation with Relationships -> Non acceptance | 0.30 | 0.21 | ─ | 0.48 |  | ** |  |  |
|  | Preoccupation with Relationships -> Goals | 0.32 | 0.13 | ─ | 0.29 |  | ** |  |  |
|  | Preoccupation with Relationships -> Impulse | 0.29 | 0.12 | ─ | 0.28 |  | ** |  |  |
|  | Preoccupation with Relationships -> Awareness | 0.14 | 0.02 | ─ | 0.22 |  | NS |  |  |
|  | Preoccupation with Relationships -> Strategies | 0.41 | 0.32 | ─ | 0.55 |  | ** |  |  |
|  | Preoccupation with Relationships -> Clarity | 0.21 | 0.04 | ─ | 0.16 |  | ** |  |  |
|  |  |  |  |  |  |  |  |  |  |
|  | Preoccupation with Relationships -> Depression | 0.12 | 0.03 | ─ | 0.31 |  | * |  |  |
|  | Non acceptance -> Depression | 0.18 | 0.08 | ─ | 0.37 |  | ** |  |  |
|  | Goals -> Depression | 0.01 | -0.23 | ─ | 0.26 |  | NS |  |  |
|  | Impulse -> Depression | 0.13 | 0.02 | ─ | 0.51 |  | * |  |  |
|  | Awareness -> Depression | -0.20 | -0.52 | ─ | -0.19 |  | ** |  |  |
|  | Strategies -> Depression | 0.26 | 0.15 | ─ | 0.55 |  | ** |  |  |
|  | Clarity -> Depression | 0.21 | 0.33 | ─ | 0.93 |  | ** |  |  |
|  | **Total Effect** | **0.34** | **0.32** | ─ | **0.64** |  | ** |  |  |
|  | Indirect Effects |  |  |  |  |  |  |  |  |
|  | **Total Indirect Effect** | **0.22** | **0.13** | ─ | **0.29** |  |  |  |  |
|  | Preoccupation with Relationships -> Non acceptance -> Depression | 0.05 | 0.01 | ─ | 0.10 |  |  |  |  |
|  | Preoccupation with Relationships -> Goals -> Depression | 0.01 | -0.03 | ─ | 0.04 |  |  |  |  |
|  | Preoccupation with Relationships -> Impulse -> Depression | 0.04 | 0.01 | ─ | 0.08 |  |  |  |  |
|  | Preoccupation with Relationships -> Awareness -> Depression | -0.03 | -0.06 | ─ | -0.003 |  |  |  |  |
|  | **Preoccupation with Relationships -> Strategies -> Depression** | **0.11** | **0.05** | ─ | **0.18** |  |  |  |  |
|  | Preoccupation with Relationships -> Clarity -> Depression | 0.05 | 0.01 | ─ | 0.08 |  |  |  |  |
